# The genomic features that affect the lengths of 5’ untranslated regions in multicellular eukaryotes

**DOI:** 10.1186/1471-2105-12-S9-S3

**Published:** 2011-10-05

**Authors:** Chun-Hsi Chen, Hsuan-Yu Lin, Chia-Lin Pan, Feng-Chi Chen

**Affiliations:** 1 Division of Biostatistics and Bioinformatics, Institute of Population Health Sciences, National Health Research Institutes, Zhunan, Miaoli County, 350 Taiwan, Republic of China; 2 Department of Life Science, National Chiao-Tung University, Hsinchu, 300 Taiwan, Republic of China; 3 Department of Dentistry, Chinese Medical University, Taichung, 404 Taiwan, Republic of China

## Abstract

**Background:**

The lengths of 5’UTRs of multicellular eukaryotes have been suggested to be subject to stochastic changes, with upstream start codons (uAUGs) as the major constraint to suppress 5’UTR elongation. However, this stochastic model cannot fully explain the variations in 5’UTR length. We hypothesize that the selection pressure on a combination of genomic features is also important for 5’UTR evolution. The ignorance of these features may have limited the explanatory power of the stochastic model. Furthermore, different selective constraints between vertebrates and invertebrates may lead to differences in the determinants of 5’UTR length, which have not been systematically analyzed.

**Methods:**

Here we use a multiple linear regression model to delineate the correlation between 5’UTR length and the combination of a series of genomic features (G+C content, observed-to-expected (OE) ratios of uAUGs, upstream stop codons (uSTOPs), methylation-related CG/UG dinucleotides, and mRNA-destabilizing UU/UA dinucleotides) in six vertebrates (human, mouse, rat, chicken, African clawed frog, and zebrafish) and four invertebrates (fruit fly, mosquito, sea squirt, and nematode). The relative contributions of each feature to the variation of 5’UTR length were also evaluated.

**Results:**

We found that 14%~33% of the 5’UTR length variations can be explained by a linear combination of the analyzed genomic features. The most important genomic features are the OE ratios of uSTOPs and G+C content. The surprisingly large weightings of uSTOPs highlight the importance of selection on upstream open reading frames (which include both uAUGs and uSTOPs), rather than on uAUGs *per se*. Furthermore, G+C content is the most important determinants for most invertebrates, but for vertebrates its effect is second to uSTOPs. We also found that shorter 5’UTRs are affected more by the stochastic process, whereas longer 5’UTRs are affected more by selection pressure on genomic features.

**Conclusions:**

Our results suggest that upstream open reading frames may be the real target of selection, rather than uAUGs. We also show that the selective constraints on genomic features of 5’UTRs differ between vertebrates and invertebrates, and between longer and shorter 5’UTRs. A more comprehensive model that takes these findings into consideration is needed to better explain 5’UTR length evolution.

## Background

The length evolution of 5’ untranslated region (5’UTR) is an important topic in the evolution of eukaryotic genomes [1-8]. On the one hand, 5’UTRs of significant lengths are evolutionarily disadvantageous because they increase the energy cost in transcription and the risk of integrating premature start codons (upstream AUGs or “uAUGs”) and unfavourable mRNA secondary structures, both of which may prevent efficient protein translation [9,10]. On the other hand, 5’UTRs may contain *cis*-regulatory elements that can modulate transcription and/or translation, which potentially can convey advantages to the carrier organisms in the face of changeable environments [8,11]. However, the changes in 5’UTR length may have only minor fitness effects in most of the cases because such changes may affect the abundance (or presence), rather than the biological functions of the affected proteins. As such, random genetic drift should play an important role in the length evolution of 5’UTR, particularly for organisms with a small effective population size. Lynch and colleagues proposed a null model for 5’UTR length evolution, which considers the gains/losses of transcription initiation signals (TISs) as a stochastic process that causes variations in 5’UTR length, and the selection against uAUGs as the major force to restrain unlimited elongation of 5’UTRs [7]. In Lynch et al’s simulation study, the model effectively depicted the skewed distributions of 5’UTR length in eukaryotes [7]. Nevertheless, Lynch et al’s model does not consider the potential influences of background genomic features. For example, by incorporating the factor of G+C content in simulation studies, Reuter and colleagues demonstrated that mutational bias also has an effect on 5’UTR length [6]. They suggested that G+C content could affect the probability of stochastic gains/losses of TIS, thus affecting the lengths of 5’UTRs. However, Reuter et al. demonstrated only a weak positive correlation between 5’UTR length and G+C content. Considering the relatively small explanatory power of Reuter et al.’s model, we hypothesize that G+C content may be only one of the many genomic features that affect 5’UTR length [6].

One important feature that may affect 5’UTR length is the presence of upstream open reading frames (uORFs). A uORF is composed of one uAUG, one in-frame stop codon downstream of the uAUG, and at least one non-stop codon in between [10]. uORFs can significantly reduce the efficiency of protein translation, and are thus potentially deleterious. In Lynch et al’s stochastic model, the selection against uAUGs is regarded as the only effect to limit 5’UTR elongation. Considering the importance of uORFs, we reason that stop codons within 5’UTRs (upstream stop codons, or “uSTOPs”) may also play an important role in affecting 5’UTR length. The influences of uSTOPs on 5’UTR length, however, have not been systematically analyzed.

In addition to uAUG and uSTOPs, certain dinucleotides may also affect 5’UTR length. For example, the highly mutable CG dinucleotides can easily change into TG (or UG in mRNA) because of methylation-induced spontaneous deamination of cytosine [12]. Such biased mutation will lead to overrepresentation of UG and CA dinucleotides in heavily methylated genomic regions. Notably, the UG dinucleotide can combine with adenine to form either a start (AUG) or a stop (UGA) codon. Therefore, the UG dinucleotides in 5’UTRs are expected to be evolutionarily constrained. Also noteworthy is that the prevalence of CG methylation in gene body differs considerably between vertebrates and invertebrates [13,14]. It is of interest to investigate whether this difference is reflected in the genomic determinants of 5’UTR length.

Aside from the abovementioned features, the dinucleotides that affect mRNA stability may also have some effects on 5’UTR length. The UA and UU dinucleotides are particularly important because both are targeted by ribonuclease for mRNA degradation [15,16] Therefore, overrepresentation of UA/UU dinucleotides may hamper the elongation of 5’UTRs. Moreover, UA is also a subsequence of the UAA stop codon. Therefore, these dinucleotides are supposedly also associated with 5’UTR length evolution.

In the study, we attempt to examine the effects of the above genomic features on the lengths of 5’UTRs. We use a multiple linear regression model to delineate the correlations between 5’UTR length and the linear combination of these features in six vertebrate (human, mouse, rat, chicken, African clawed frog, and zebrafish) and four invertebrate species (fruit fly, mosquito, sea squirt, and nematode). We find that the linear combination of these genomic features can explain a significant proportion of the length variations of 5’UTRs in the ten examined species. Furthermore, the relative contributions of the genomic features differ among lineages, suggesting a potential role of lineage-specific genomic features in the evolution of 5’UTRs. Intriguingly, in all of the ten examined species, uSTOPs play a more important role than uAUGs, which differs from the well recognized concept that uAUGs play a dominant role in 5’UTR evolution. Our study thus brings new insights into the length evolution of 5’UTR in multi-cellular eukaryotes.

## Methods

### Sequences of 5’ untranslated regions

The sequences of 5’UTRs were retrieved from UTRdb (http://utrdb.ba.itb.cnr.it/; updated in July 2010), which harboured the sequences and annotations of experimentally validated 5’UTRs [17]. The species with relatively abundant 5’UTR information were selected, including six vertebrates – human (*Homo sapiens*), mouse (*Mus musculus*), rat (*Rattus norvegicus*), chicken (*Gallus gallus*), African clawed frog (*Xenopus tropicalis*), and zebrafish (*Danio rerio*), and four invertebrates – fruit fly (*Drosophila melanogaster*), mosquito (*Anopheles gambiae*), sea squirt (*Ciona intestinalis*), and nematode (*Caenorhabditis elegans*). Only the genes with experimentally validated protein products were retained. In case of alternative splicing, one transcript was randomly selected to avoid overweighting of certain genes.

### The observed-to-expected ratio of tri- and di-nucleotides

The observed-to-expected (OE) ratios of tri- and di-nucleotides were derived to measure the strength of selection pressure on these genomic components. The OE ratio is simply the observed number of tri- or di-nucleotides divided by its expected number. The expected number of tri- or di-nucleotides was calculated as follows [14]:(1)

where *f_i_* is the frequency of nucleotide *i* (A, U, C, or G), and *L* is the length of the sequence of interest. For example, the expected number of AUG equals to *L* × *f_A_* × *f_U_* × *f_G_*.

### Construction of linear regression models

All the statistical analyses were performed by using the R program (http://www.r-project.org). The multiple linear regression model is as follows:(2)

where *Y* stands for the 5’UTR length (log 10 scale), variables *X_i_* include the G+C content, the OE ratios of trinucleotides AUG, UGA, UAA, UAG, and dinucleotides CG, UG, UU, and UA.

A standard procedure for model selection was used to exclude the genomic features that did not significantly associate with 5’UTR length [18]. To evaluate whether collinearity between genomic features may affect our model, the variance inflation factors (VIF) of each feature were calculated for each species. The VIF measures the increase of the variance of a genomic feature due to its dependency on other features. If a feature has a VIF larger than 10, the correlation between the dependent variable (5’UTR length) and the feature is suggested to depend on other genomic features. In this case, it is difficult to evaluate the effects of individual features on 5’UTR length. Since the VIF in our models were all smaller than 10 (Additional file 1), collinearity between genomic features did not appear to affect our models.

To construct the regression model, we first examined the genomic features to be analyzed in our 5’UTR dataset. We found that approximately half of the 5’UTRs had lengths smaller than 160 bp, and that more than half of the 5’UTRs had at least one zero-OE ratio of trinucleotide. To minimize the number of such non-informative entries, we excluded the 5’UTRs with more than one zero-OE ratios.

To measure the relative importance of each genomic feature, the relative contribution to variability explained (RCVE) was calculated [19]. An RCVE was calculated as follows:

(3),

where  and  stand for the R^2^ value of the full model (which includes all of the analyzed genomic features) and in a reduced model, respectively. A reduced model was established by removing one genomic feature of interest. A large RCVE indicates a significant contribution of the genomic feature of interest to the regression model [19].

### The minimal length of a sequence for a specific trinucleotide to occur by chance

The minimal length of a sequence for a specific type of trinucleotide to occur by chance can be easily estimated by replacing the left half of equation (1) with unity. In other words, the minimal length is the reciprocal of . For example, the minimal length of a sequence for an AUG trinucleotide to occur by chance is 1/(*f_A_* × *f_U_* × *f_G_*).

## Results

### Approximately 14%~33% of the length variations of 5’UTRs can be explained by the underlying genomic features

We selected ten well-studied animal species for comparison, including six vertebrates and four invertebrates (see Methods). The genomic features examined here include G+C content, the OE ratios of AUG, UGA, UAA, and UAG trinucleotides, and the OE ratios of CG, UG, UA, and UU dinucleotides. The OE ratio measures whether the frequency of a specific dinucleotide or trinucleotide deviates from expectation. If the OE ratio is close to unity, the observed di- or tri-nucleotides may have occurred simply by chance. In other words, these di- or trinucleotides are likely subject to neutral selection. We used these measurements as candidate predictors to establish a multiple regression model to predict 5’UTR length in the ten examined species. The backward model selection approach was employed to remove candidate genomic features that do not associate significantly with 5’UTR length. The VIFs were calculated for each species to examine whether collinearity existed among the examined genomic features. Our results show that collinearity does not exist between any pair of predictors (all VIF < 10, Additional file 1) despite the fact that some of the genomic features are correlated (e.g. UG and AUG). Accordingly, we can investigate how individual genomic features correlate with 5’UTR length.

The R^2^ values of the models fall between 0.14 and 0.33, indicating a good explanatory power for within-species 5’UTR length distribution in all of the studied species (Table 1). Interestingly, the invertebrate regression models have higher R^2^ values (0.23~0.33) than those of vertebrates (0.14~0.21), suggesting that the linear combination of the selected genomic features can explain 5’UTR length better for invertebrates than for vertebrates. Among the statistically significant determinants of 5’UTR length, the G+C content and the OE ratios of the four trinucleotides (uAUG and uSTOPs) are shared by all of the ten analyzed species. By contrast, whether the OE ratios of dinucleotides are good predictors of 5’UTR length appears to be lineage-dependent (Table 1). The most widely applicable dinucleotide predictor is the UG dinucleotide, which is shared by all of the studied species except for the African clawed frog.

**Table 1:** Coefficients of linear regression models for 5’UTR length prediction

Predictors	human	mouse	rat	chicken	frog	zebrafish	fruit fly	mosquito	sea squirt	nematode
G+C content	0.53	0.56	0.58	0.65	0.58	0.55	1.65	1.23	1.62	0.89
AUG OE	-0.01	-0.03	-0.01	-0.04	-0.04	-0.03	-0.02	-0.08	-0.04	-0.07
UGA OE	-0.06	-0.07	-0.08	-0.05	-0.09	-0.08	-0.15	-0.10	-0.12	-0.10
UAA OE	-0.05	-0.06	-0.08	-0.05	-0.10	-0.11	-0.12	-0.12	-0.17	-0.22
UAG OE	-0.13	-0.13	-0.15	-0.14	-0.13	-0.12	-0.20	-0.15	-0.12	-0.17
CG OE	-0.03	0.04	ns^a^	-0.08	-0.10	0.02	ns	-0.05	ns	-0.02
UG OE	-0.05	-0.03	-0.04	-0.10	ns	-0.04	0.12	0.07	0.10	-0.04
UU OE	0.04	0.02	ns	ns	0.04	ns	0.09	ns	0.17	ns
UA OE	-0.03	-0.04	-0.04	ns	ns	ns	0.18	0.11	ns	ns
Adjusted R^2^	0.14	0.15	0.16	0.21	0.19	0.14	0.29	0.23	0.33	0.30

^a^ns, not significant

### Associations between 5’UTR length and individual genomic features

The correlations between 5’UTR length and five genomic features (G+C content, uAUG OE, and the OE ratios of uSTOPs) are consistent across all species (Table 1): G+C content is positively correlated with 5’UTR length, whereas all of the other four genomic features show a negative correlation. There are two possible reasons for the positive correlation between G+C content and 5’UTR length. First, genes with AT-rich TISs tend to have longer 5’UTRs in G+C-rich genomic regions because it is difficult to find an alternative TIS in such a region once the original TIS is disrupted [6]. Second, a higher G+C content leads to a lower probability of random occurrence of AUGs. The decreased number of uAUGs may have allowed the stochastic extension of 5’UTRs [7]. Meanwhile, among the four OE ratios of dinucleotides, only the OE ratio of UU shows a consistently positive correlation with 5’UTR length across multiple species (Table 1), although the reason for the correlation remains unclear.

### G+C% and selection on uSTOPs contribute most to the variations in 5’UTR length

**Figure 1:**
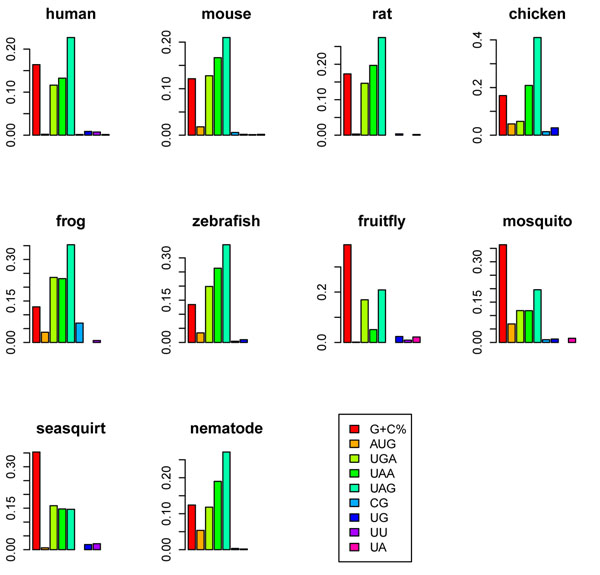
**The relative contributions to variability explained (RCVE) of different genomic features in the analyzed species.** The RCVE was calculated according to the difference of R^2^ between the full model (with all predictors) and the reduced model (remove one predictor of interest). A large RCVE indicates a large contribution of a specific predictor.

To evaluate the extent each genomic feature affects the 5’UTR length, we calculated the relative contribution to variability explained (RCVE, see Methods) [19]. As shown in Figure 1, G+C content is the dominant determinant in three of the four studied invertebrates (fruit fly, mosquito, and sea squirt), whereas UAG OE is the most influential determinant in all of the six vertebrates and nematode. Similar results can be seen in the partial correlation analyses, where the correlation between 5’UTR length and each individual feature is evaluated while the other genomic features are controlled (Table 2). Surprisingly, the selection on uAUGs (AUG OE ratio) appears to play a relatively minor role in affecting 5’UTR length. Meanwhile, the surprisingly large RCVEs of uSTOP OE ratios suggest the importance of these trinucleotides in 5’UTR length evolution (Figure 1). Considering that uAUGs and uSTOPs together can form uORFs, our observation seems to imply that the major target of selection in 5’UTRs is likely uORFs, rather than uAUGs *per se*. Indeed, we found that the presence/absence of uORFs is significantly correlated with 5’UTR length (Additional file 2). We have also tried to include the information of secondary structure by adding into the regression the number of G-quadruplexes (predicted by Quadparser [20]). However, G-quadruplexes have only small effects on 5’UTR length (Additional file 3).

**Table 2:** The coefficients in the partial correlations between 5’UTR length and each predictor while other genomic features are controlled.

Predictors	human	mouse	rat	chicken	frog	zebrafish	fruit fly	mosquito	sea squirt	nematode
G+C content	0.160	0.148	0.143	0.211	0.170	0.146	**0.366**	**0.317**	**0.389**	0.226
AUG OE	-0.023	-0.059	ns	-0.116	-0.090	-0.074	-0.026	-0.145	-0.075	-0.145
UGA OE	-0.135	-0.152	-0.163	-0.125	-0.209	-0.176	-0.252	-0.188	-0.262	-0.218
UAA OE	-0.144	-0.173	-0.187	-0.201	-0.213	-0.186	-0.141	-0.187	-0.262	-0.244
UAG OE	**-0.187**	**-0.193**	**-0.215**	**-0.253**	**-0.235**	**-0.207**	-0.272	-0.239	-0.267	**-0.290**
CG OE	ns^a^	0.035	ns	-0.069	-0.122	0.028	ns	-0.059	ns	-0.043
UG OE	-0.039	-0.024	ns	-0.105	ns	-0.034	0.092	0.062	ns	ns
UU OE	0.036	ns	ns	ns	0.044	ns	0.061	ns	0.125	ns
UA OE	ns	-0.023	ns	ns	ns	ns	0.093	0.069	ns	ns

^a^ns, not significant.

^b^The bold-faced values represent the highest (absolute value) correlation in each specie

## Discussion

We have demonstrated that genomic features can explain 14%~33% of the length variation of 5’UTR in multi-cellular animals. These genomic features include the G+C content, the OE ratios of uAUG, uSTOPs, and CG, UG, UU, and UA dinucleotides. The RCVE analysis demonstrates that the most predominant determinants of 5’UTR length common to vertebrates and invertebrates (except for nematode) are the G+C content and the OE ratios of uSTOPs. The effects of dinucleotides appear to be weaker than trinucleotides. This observation implies that dinucleotide-related biological consequences (such as CpG methylation, mutational biases towards AT, and UU/UA-associated destabilization of mRNA) may have only minor effect on 5’UTR evolution.

There are a few limitations in the study. First, in our analysis, we excluded 5’UTRs with insufficient information. The 5’UTRs with zero-OE ratios are excluded because we are not sure whether the zero-OE ratios are a consequence of selection or they occur simply by chance (because of the short length of 5’UTR). Therefore, our linear regression model is applicable mainly for longer 5’UTRs. The lengths of shorter 5’UTRs may have been affected more by random processes (see the discussion below). Note that we have tried to exclude 5’UTRs shorter than a certain length threshold (e.g. 30 Bp). However, this approach actually skews the distribution of 5’UTR length away from normality (Additional file 4), which may compromise the applicability of the linear regression analysis. Therefore, excluding 5’UTRs with zero-OE ratios appears to be more suitable for this study.

Second, we do not take into consideration the influences of neighbouring genomic regions. For example, the G+C content in the intergenic region upstream of a 5’UTR could affect the probability of stochastic 5’UTR elongation when the TIS (can be either G+C-rich or A+ T-rich) is somehow disrupted [7]. Therefore, the sequence motif of TIS and the G+C content upstream of a 5’UTR may together affect its length. Unfortunately, TISs remain unknown for a large fraction of genes, which has restricted researchers from addressing this important issue.

In spite of the above limitations, the study has demonstrated that 5’UTR length in multi-cellular eukaryotes are affected by certain genomic features, in addition to random genetic drift and selection against uAUGs. Since many eukaryotic species have a very small effective population size, the influence of random genetic drift plays a major role for the evolution of sequences whose mutations usually have weak fitness effect (e.g. 5’UTR) [7,21]. Nevertheless, the selection on certain genomic compositions remains important. One of the most influential genomic features is uAUG. This type of trinucleotide can disturb normal translation and significantly reduce protein abundance [10]. Interestingly, our analysis shows that an upstream stop codon (UAG) plays an even more important role in this regard. This observation seems to suggest that the selection target is uORF (or the potential to form uORF) but not uAUG *per se*, for a uSTOP is an indispensable part of a uORF. Furthermore, a uAUG may have different levels of fitness effects when it is incorporated in different types of uORFs. In the case of strictly upstream uORF (a uORF that is located entirely within a 5’UTR), a uAUG probably will cause reduced protein production of the main coding sequence (CDS) [10]. By contrast, in the case of overlapping uORF (a uORF with its uAUG in 5’UTR but its stop codon located within the downstream CDS), the translation that begins at the uAUG may cause skipping of the main start codon, and therefore, complete inhibition of normal protein translation or production of N-truncated proteins [5]. Both of the possible outcomes can be strongly deleterious. Generally, overlapping uORFs occur less frequently than strictly upstream uORFs [22]. Therefore, although uAUGs not incorporated in strictly upstream uORFs may turn out to be part of overlapping uORFs (and thus have a strong effect on translation), collectively they may have a smaller effect on 5’UTR length evolution than strictly upstream uORFs. An alternative explanation for the importance of uSTOPSs is that since overlapping uORFs are in general more deleterious than strictly upstream uORFs, uSTOPs may be favoured by selection because they can potentially prevent uORFs from extending into coding sequences.

Another common determinant is the G+C content, which is the most important determinant of 5’UTR length in all of the studied invertebrate species except for nematode. In comparison, for vertebrates, the influence of G+C content is second to uSTOPs. Since the G+C content is related to the stochastic elongation of 5’UTRs after the disruption of TISs [7], we speculate that this vertebrate-invertebrate divergence may have resulted from the difference in the G+C content of the commonly used TISs between these two groups of organisms. For instance, TATA box (an AT-rich regulatory element), the best characterized transcription factor binding site (TFBS), appears to be used with different frequencies between vertebrates and invertebrates [23,24]. If such A+T-rich TFBSs are used for transcriptional initiation, a G+C-rich genomic context will lead to an increased level of 5’UTR elongation (as compared with an A+T-rich context) once the TFBSs are disrupted by mutations [6]. However, we do not know the exact proportions of A+T- and G+C-rich TFBSs in the studied species. Therefore, the real cause of the vertebrate-invertebrate difference in the determinants of 5’UTR length remains an open question.

Note that the lengths of 5’UTRs in multicellular eukaryotes are determined by two major driving forces – the stochastic elongation due to reduced effective population size (for which G+C content is more important), and the selection against deleterious genomic features in longer 5’UTRs (for which the OE rations of uAUGs and uSTOPs are more important). Interestingly, the G+C content and 5’UTR length may actually affect the expected numbers of the trinucleotides. To highlight this point, we calculated the minimal length of a sequence in which one specific type of trinucleotide can be observed given a certain G+C content (Methods, Additional file 5). If a 5’UTR has a length smaller than the minimal length, the selection on a certain trinucleotide is supposed to be less effective because the probability of the trinucleotide to occur by chance is very small. Since the trinucleotides examined in the study are mostly G+C-poor, the minimal lengths are positively correlated with the G+C content in most of the cases (Additional file 6). Furthermore, such minimal lengths for vertebrates are on average longer than for invertebrates because the former generally have higher G+C genomic contents. Taken another way, the “length threshold” for the natural selection on the trinucleotides to be effective is higher for vertebrates than for invertebrates. Our results also imply that 5’UTRs of different lengths are subject to different evolutionary forces, with longer 5’UTRs more likely affected by selection, and shorter 5’UTRs by the stochastic process (or genetic drift).

## Conclusions

The length evolution of 5’ UTRs is an important topic in the evolution of eukaryotic genomes. It was previously proposed that genetic drift and selection on uAGUs were the major determinants of 5’UTR length. Here we add new perspectives to this topic by demonstrating that (1) vertebrates and invertebrates have subtle differences in genomic features that affect 5’UTR length; (2) genomic features other than uAUGs, particularly uSTOPs, play an important role in the length evolution of 5’UTR; and (3) shorter and longer 5’UTRs are subject to distinct evolutionary forces. A more complicated model that takes these observations into consideration is thus required to better explain the length evolution of 5’UTRs.

## Competing interests

The authors declare that they have no conflict of interests.

## Authors' contributions

CHC and FCC conceived the study. CHC, HYL, and CLP analyzed the data. CHC and FCC interpreted the results and drafted the manuscript. All authors have read and agreed on the manuscript.

## Supplementary Material

Additional file 1 **The variance inflation factors (VIFs) of each genomic feature in the linear regression models for 5’UTR length prediction.** All of the VIFs are smaller than 10, indicating that the collinearity between the analyzed variables is negligible. Click here for file

Additional file 2 **Evaluation of the effect of uORF presence/absence on 5’UTR length.** The regression model was: Y = β_0_ + β_1_*X_1_ +β_2_*X_2_ +β_3_*X_3_ +β_4_*X_4_ +β_5_*X_5_ +β_6_*X_6_ +β_7_*X_7_ +β_8_*X_8_ +β_9_*X_9_ +β_10_*X_10_ + ε Y: Log_10_5’UTR length; X_1_: GC content; X_2_: AUG_O/E_; X_3_: UGA_O/E_; X_4_: UAA_O/E_; X_5_: UAG_O/E_; X_6_: CpG_O/E_; X_7_: UpG_O/E_; X_8_: UpU_O/E_; X_9_: UpA_O/E_; X_10_: uORF presence (1) / absence (0). Click here for file

Additional file 3 **The relative contributions to variability explained (RCVE) of different genomic features in the analyzed species.** In this figure, the number of G-quadruplexes is included in the multiple regression analysis and analyzed for RCVE. G-quadruplexes actually contribute to only a small proportion of 5’UTR length variability. Click here for file

Additional file 4 **The Q-Q plot of 5’UTR length distribution for (A) the datasets analyzed in Table**1**; (B) the datasets where 5’UTRs shorter than 30 Bp were excluded.** Note that at the lower left corner in (B), the data points skew seriously from normality as compared with (A). Click here for file

Additional file 5**The minimal length of a sequence for a specific trinuelceotide to occur at least once in the ten analyzed organisms.** The minimal length was measured as  (see Methods for more details).Click here for file

Additional file 6 **The correlation between G+C content and the minimal length for a specific trinuelceotide to occur at least once.** "*R*" stands for the Pearson's coefficient of correlation. "*P*" is the p-value of the linear regression model. Click here for file
