# Supplementary material for: The genomic features that affect the lengths of 5’ untranslated regions in multicellular eukaryotes
Source: BMC Bioinformatics. 2011 Oct 5;12(Suppl 9):S3. doi: 10.1186/1471-2105-12-S9-S3 (PMC3283318; doi:10.1186/1471-2105-12-S9-S3)
Supplement: Additional file 1 — The variance inflation factors (VIFs) of each genomic feature in the linear regression models for 5’UTR length prediction. All of the VIFs are smaller than 10, indicating that the collinearity between the analyzed variables is negligible. [file 1471-2105-12-S9-S3-S1.pdf]

Additional file 1. The variance inflation factors (VIFs) of each feature in the linear regression models for 5'UTR length prediction

Predictors	human	mouse	rat	chicken	frog	zebrafish	fruit fly	mosquito	sea squirt	nematode
G+C %	1.81	1.85	1.56	1.97	1.09	1.13	1.08	1.03	1.06	1.07
AUG OE	1.36	1.20	1.22	1.45	1.25	1.32	1.24	1.14	1.25	1.49
UGA OE	1.39	1.25	1.26	1.43	1.30	1.41	1.22	1.16	1.32	1.50
UAA OE	1.46	1.46	1.40	1.59	1.30	1.37	1.28	1.26	1.31	1.31
UAG OE	1.84	1.82	1.76	2.05	1.61	1.48	1.43	1.45	1.47	1.43
CG OE	2.22	2.20	1.81	2.08	1.25	1.27	1.17	1.16	1.26	1.21
UG OE	2.12	1.89	1.89	2.06	1.90	2.08	1.67	1.59	1.98	2.06
UU OE	1.28	1.29	1.27	1.40	1.39	1.33	1.29	1.31	1.63	1.28
UA OE	2.28	2.19	2.10	2.43	2.02	2.06	1.86	1.80	1.92	1.81
