# Supplementary material for: The genomic features that affect the lengths of 5’ untranslated regions in multicellular eukaryotes
Source: BMC Bioinformatics. 2011 Oct 5;12(Suppl 9):S3. doi: 10.1186/1471-2105-12-S9-S3 (PMC3283318; doi:10.1186/1471-2105-12-S9-S3)
Supplement: Additional file 2 — Evaluation of the effect of uORF presence/absence on 5’UTR length. The regression model was: Y = β0 + β1*X1 +β2*X2 +β3*X3 +β4*X4 +β5*X5 +β6*X6 +β7*X7 +β8*X8 +β9*X9 +β10*X10 + ε Y: Log105’UTR length; X1: GC content; X2: AUGO/E; X3: UGAO/E; X4: UAAO/E; X5: UAGO/E; X6: CpGO/E; X7: UpGO/E; X8: UpUO/E; X9: UpAO/E; X10: uORF presence (1) / absence (0). [file 1471-2105-12-S9-S3-S2.pdf]

Additional file 2. Evaluation of the effect of uORF presence/absence on 5'UTR length. The presence/absence of uORF was added into the multiple regression model, and the statistical significance of the coefficient (β_{10}) of this newly added variable (X_{10}) was evaluated.

Species	Estimate of β_{10}	*t* value	*p* value
Human(*Homo sapiens*)	0.259	39.176	< 2e-16 ***
Mouse(*Mus musculus*)	0.276	41.105	< 2e-16 ***
Rat(*Rattus norvegicus*)	0.281	31.794	<2e-16 ***
Frog(*Xenopus tropicalis*)	0.269	33.064	< 2e-16 ***
Chicken(*Gallus gallus*)	0.234	12.492	< 2e-16 ***
Zebrafish(*Danio rerio*)	0.250	40.752	< 2e-16 ***
Mosquito(*Anopheles gambiae*)	0.243	24.415	< 2e-16 ***
Fruit fly(*Drosophila melanogaster*)	0.324	43.084	< 2e-16 ***
Seasquirt(*Ciona intestinalis*)	0.200	6.956	1.33e-11 ***
Nematode(*Caenorhabditis elegans*)	0.300	25.096	<2e-16 ***

The regression model was:

$Y = \beta_0 + \beta_1*X_1 +\beta_2*X_2 +\beta_3*X_3 +\beta_4*X_4 +\beta_5*X_5 +\beta_6*X_6 +\beta_7*X_7 +\beta_8*X_8 +\beta_9*X_9 +\beta_{10}*X_{10} + \varepsilon$

Y : Log_{10}5'UTR length
X1 : GC content
X2 : $AUG_{O/E}$
X3 : $UGA_{O/E}$
X4 : $UAA_{O/E}$
X5 : $UAG_{O/E}$
X6 : $CpG_{O/E}$
X7 : $UpG_{O/E}$
X8 : $UpU_{O/E}$
X9 : $UpA_{O/E}$
X10 : uORF presence (1) / absence (0)
