# Supplementary figures and images for: The genomic features that affect the lengths of 5’ untranslated regions in multicellular eukaryotes
Source: BMC Bioinformatics. 2011 Oct 5;12(Suppl 9):S3. doi: 10.1186/1471-2105-12-S9-S3 (PMC3283318; doi:10.1186/1471-2105-12-S9-S3)

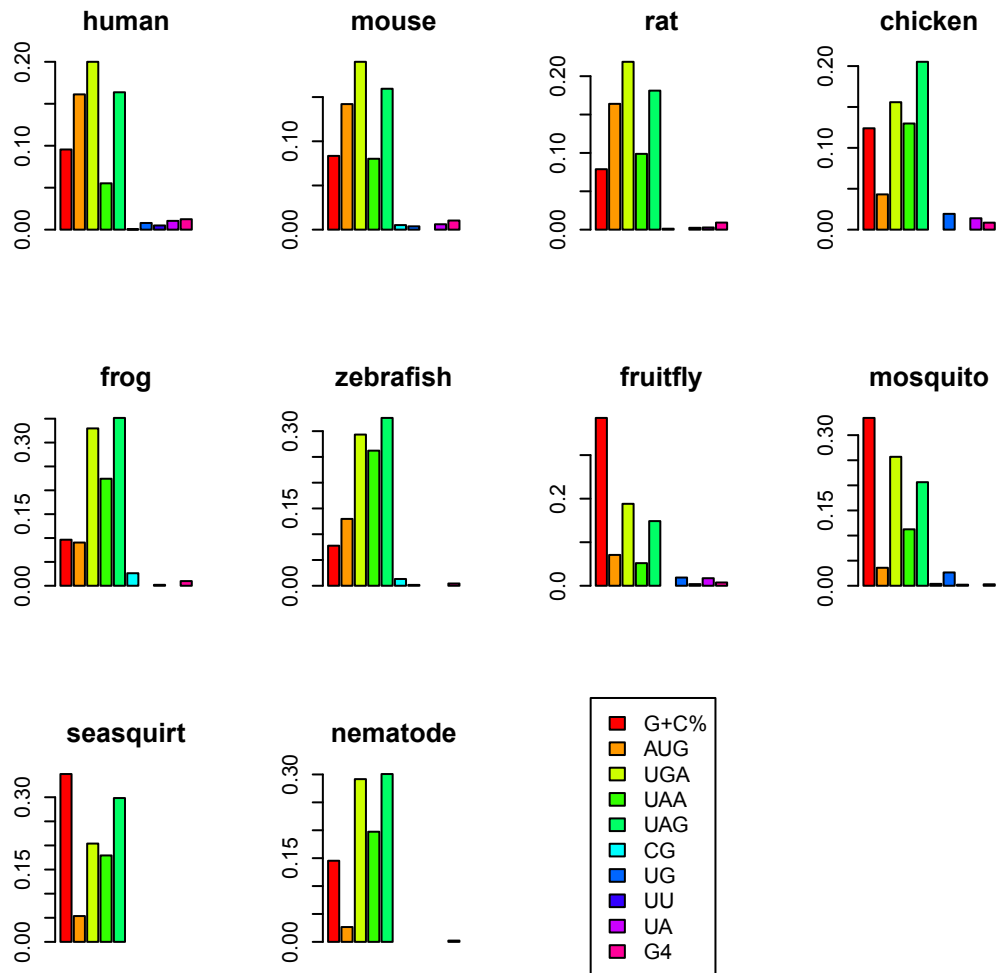

Supplement: Additional file 3 — The relative contributions to variability explained (RCVE) of different genomic features in the analyzed species. In this figure, the number of G-quadruplexes is included in the multiple regression analysis and analyzed for RCVE. G-quadruplexes actually contribute to only a small proportion of 5’UTR length variability. [file 1471-2105-12-S9-S3-S3.pdf]

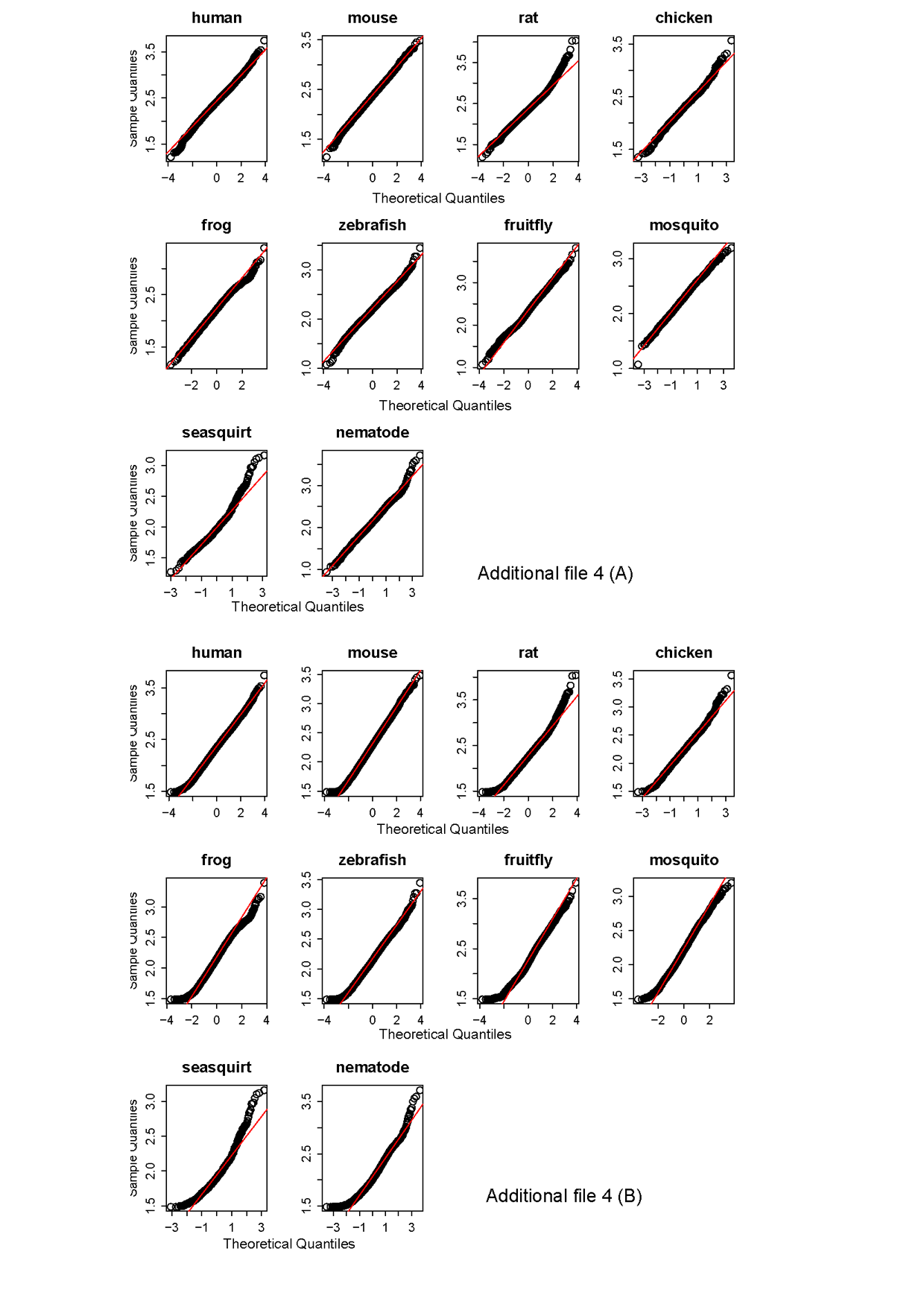

Supplement: Additional file 4 — The Q-Q plot of 5’UTR length distribution for (A) the datasets analyzed in Table1; (B) the datasets where 5’UTRs shorter than 30 Bp were excluded. Note that at the lower left corner in (B), the data points skew seriously from normality as compared with (A). [file 1471-2105-12-S9-S3-S4.tiff]

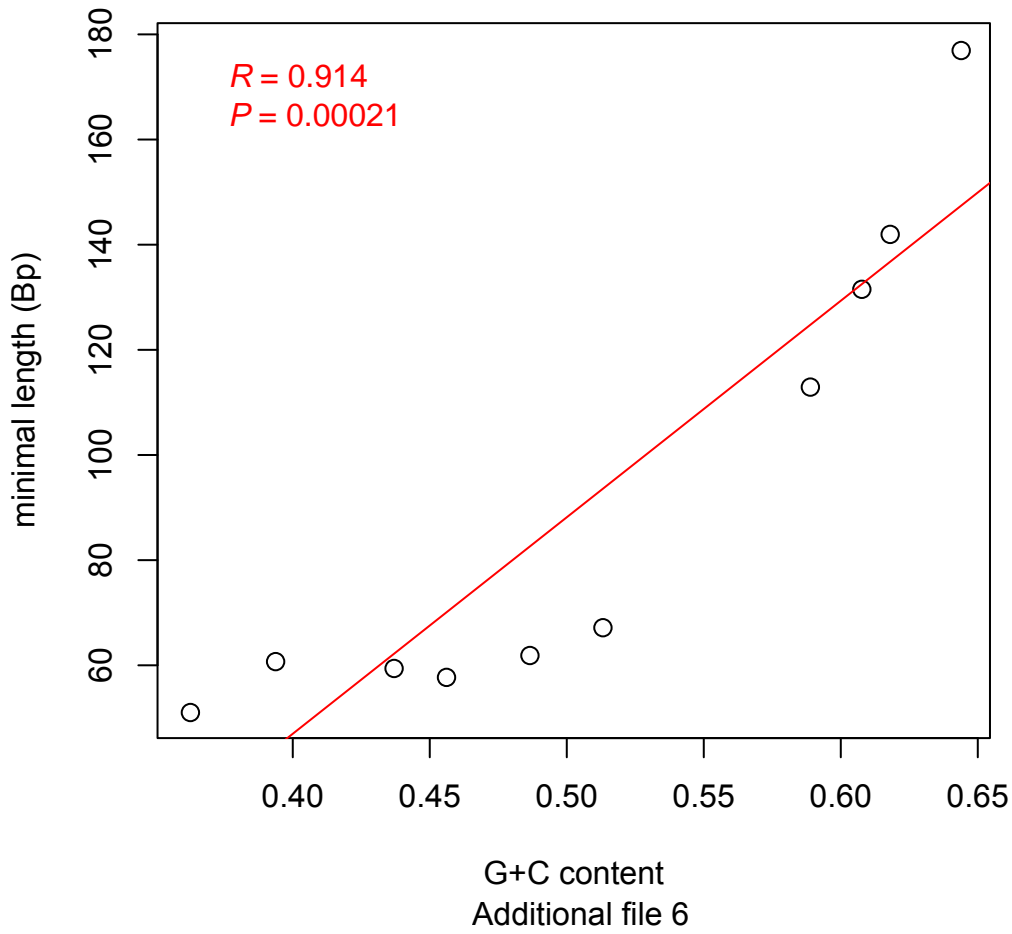

Supplement: Additional file 6 — The correlation between G+C content and the minimal length for a specific trinuelceotide to occur at least once. "R" stands for the Pearson's coefficient of correlation. "P" is the p-value of the linear regression model. [file 1471-2105-12-S9-S3-S6.pdf]
