# Supplementary material for: The genomic features that affect the lengths of 5’ untranslated regions in multicellular eukaryotes
Source: BMC Bioinformatics. 2011 Oct 5;12(Suppl 9):S3. doi: 10.1186/1471-2105-12-S9-S3 (PMC3283318; doi:10.1186/1471-2105-12-S9-S3)
Supplement: Additional file 5 — The minimal length of a sequence for a specific trinuelceotide to occur at least once in the ten analyzed organisms. The minimal length was measured as (see Methods for more details). [file 1471-2105-12-S9-S3-S5.pdf]

Additional file 5. The minimal length of a sequence for a specific trinucleotide to occur at least once in the ten analyzed organisms.

species	A%	T%	G%	Min L (Bp) AUG/UAG/UGA	Min L (Bp) UAA
human	19.21%	19.06%	31.29%	87.3	142.2
mouse	19.66%	19.65%	30.73%	84.2	131.7
rat	20.86%	20.33%	29.68%	79.4	113.0
chicken	17.74%	17.94%	33.35%	94.2	177.1
frog	25.01%	23.74%	26.77%	62.9	67.3
zebrafish	27.90%	26.57%	23.32%	57.8	48.4
fruit fly	31.51%	24.86%	21.43%	59.6	40.5
mosquito	26.90%	24.52%	24.44%	62.0	56.4
sea squirt	32.90%	30.91%	19.21%	51.2	29.9
nematode	30.77%	29.94%	17.84%	60.8	35.3

The trinucleotides analyzed in the study include AUG, UGA, UAG, and UAA. Note that the former three have the same nucleotide composition.
